# Supplementary material for: QSAR, ADMET In Silico Pharmacokinetics, Molecular Docking and Molecular Dynamics Studies of Novel Bicyclo (Aryl Methyl) Benzamides as Potent GlyT1 Inhibitors for the Treatment of Schizophrenia
Source: Pharmaceuticals (Basel). 2022 May 27;15(6):670. doi: 10.3390/ph15060670 (PMC9228289; doi:10.3390/ph15060670)
Supplement: Supplementary file 1 [file pharmaceuticals-15-00670-s001.zip › Supplemantary data.pdf]

Table S1: The forty-four molecules and their biological activities.

N°	Structure	IC_{50}	$Log_{10}IC_{50}$	N°	Structure	IC_{50}	$Log_{10}IC_{50}$	N°	Structure	IC_{50}	$Log_{10}IC_{50}$
1	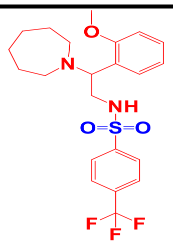	37	1,568202	9	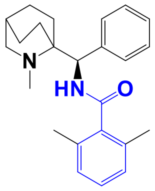	1	0	17	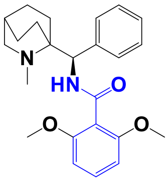	6	0,778151
2	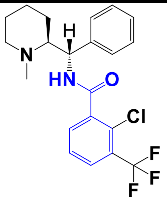	3	0,477121	10	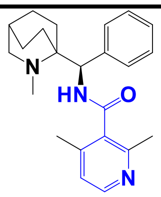	494	2,69373	18	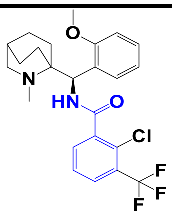	340	2,531479
3	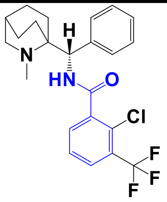	41	1,612784	11	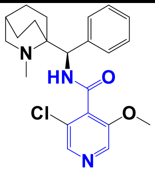	474	2,67578	19	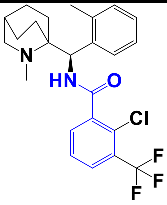	514	2,710963
4	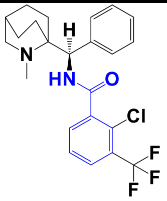	6	0,778151	12	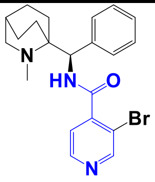	75	1,87506	20	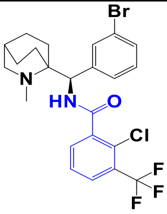	9	0,954242
5	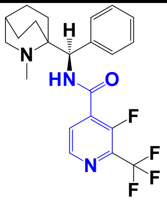	92	1,963788	13	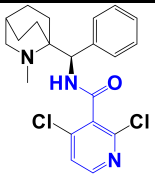	809	2,90795	21	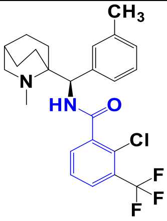	29	1,462398
6	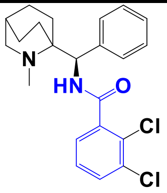	1	0	14	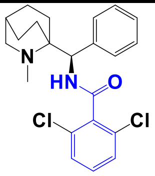	23	1,36173	22	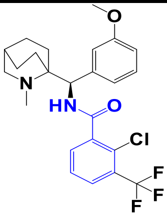	177	2,247973
7	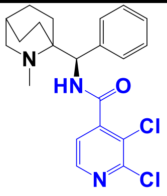	6	0,778151	15	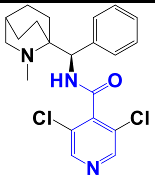	45	1,65321	23	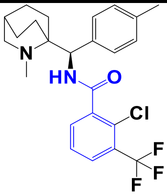	1703	3,231215

8	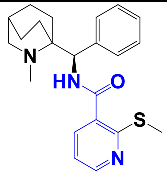	3	0,477121	16	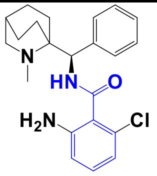	8	0,90309	24	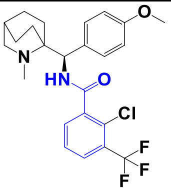	1759	3,245266
25	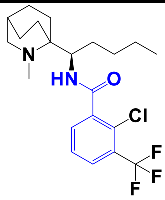	3362	3,526598	32	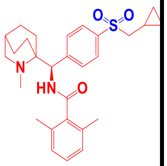	1	0	39	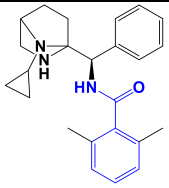	2	0,301030
26	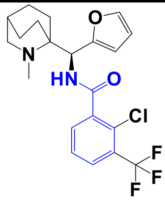	78	1,892095	33	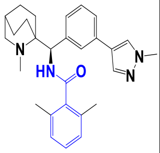	946	2,97589	40	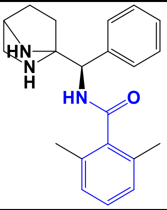	58	1,763428
27	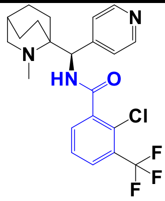	18	1,255272	34	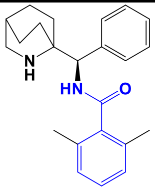	3	0,47712	41	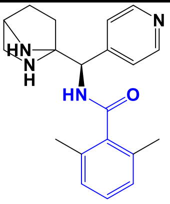	45	1,653212
28	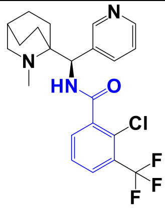	211	2,324282	35	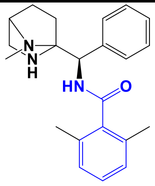	3	0,47712	42	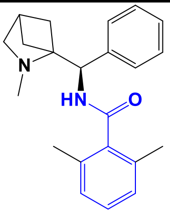	7	0,845098
29	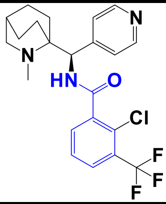	904	2,956168	36	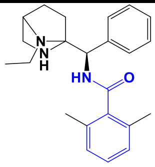	4	0,60206	43	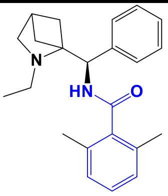	3	0,477121
30	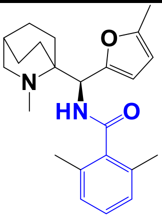	1	0	37	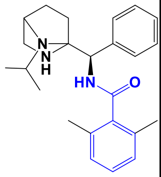	1	0	44	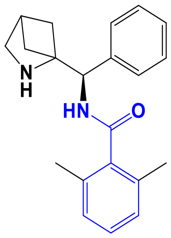	168	2,225309
31	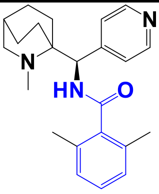	1	0	38	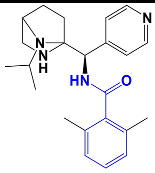	10	1				

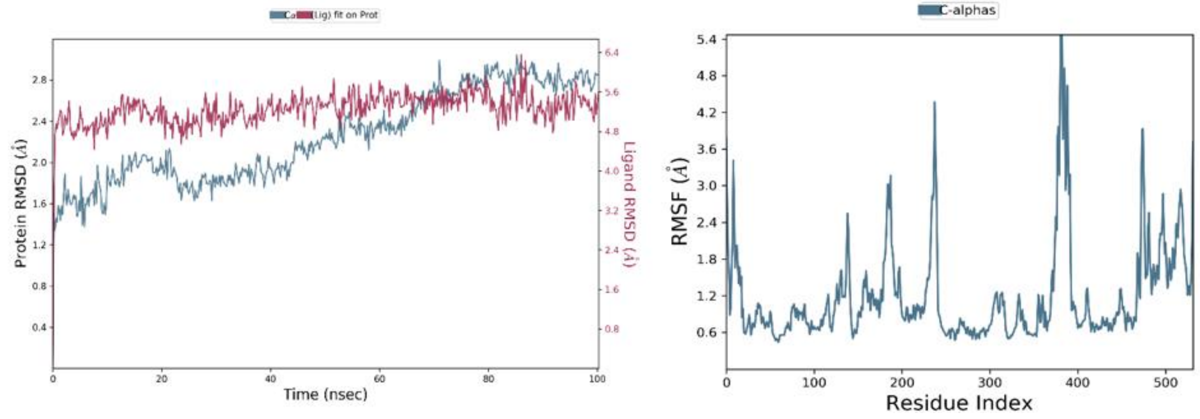

C1

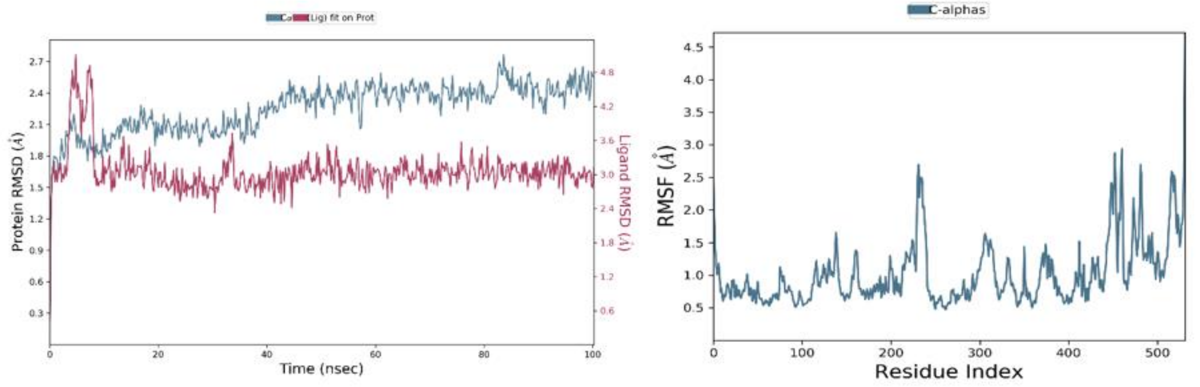

C3

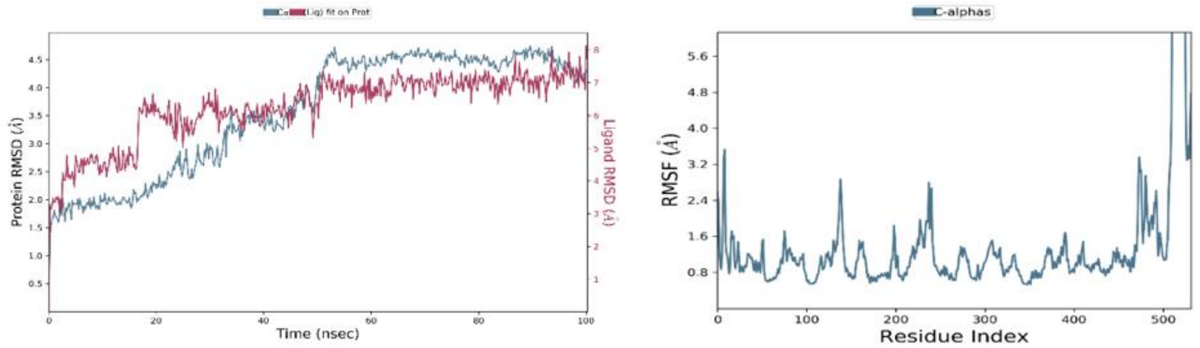

C4

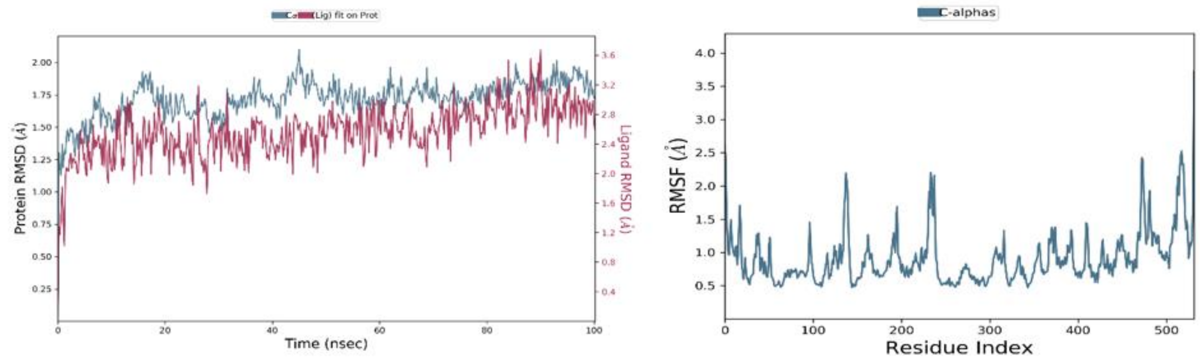

C5

Figure S1: RMSD and RMSF graphs for L6, L30, L31, L37 ligands complexed with the dopamine transporter membrane protein during 100 ns.

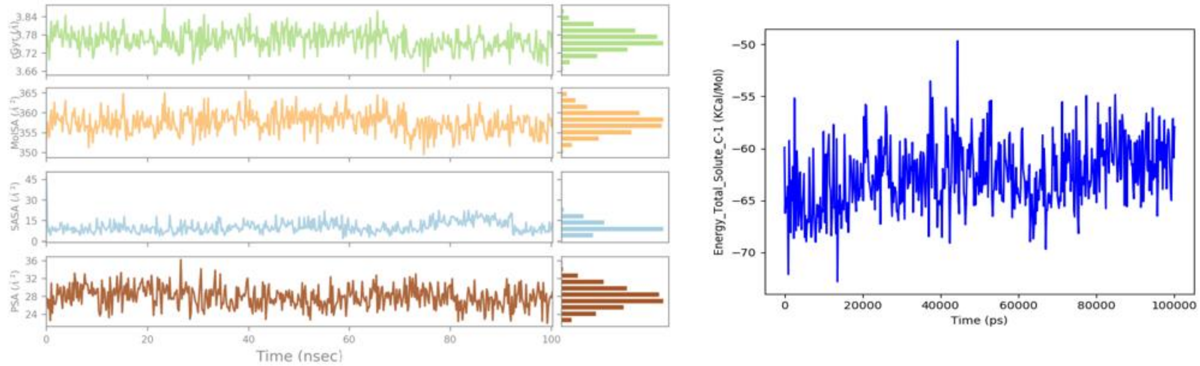

C1

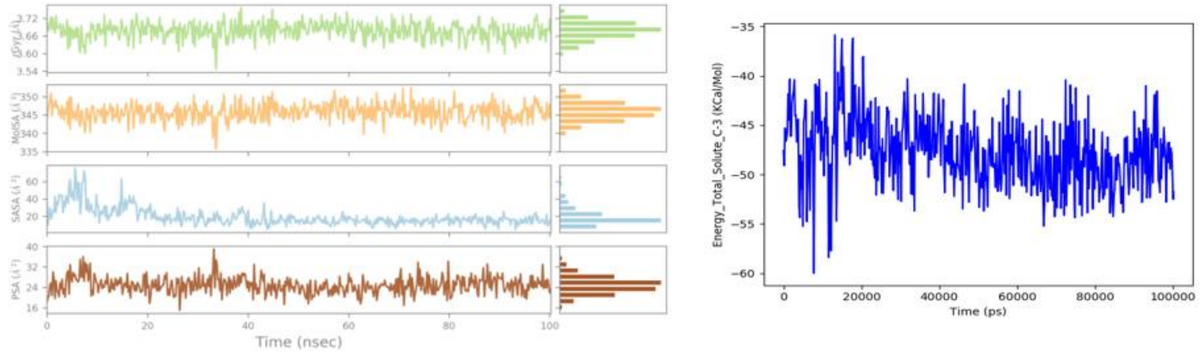

C3

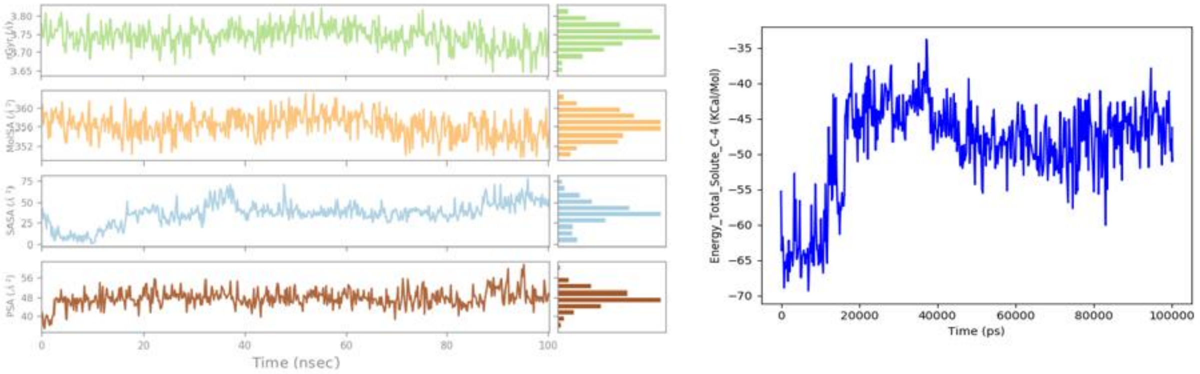

C4

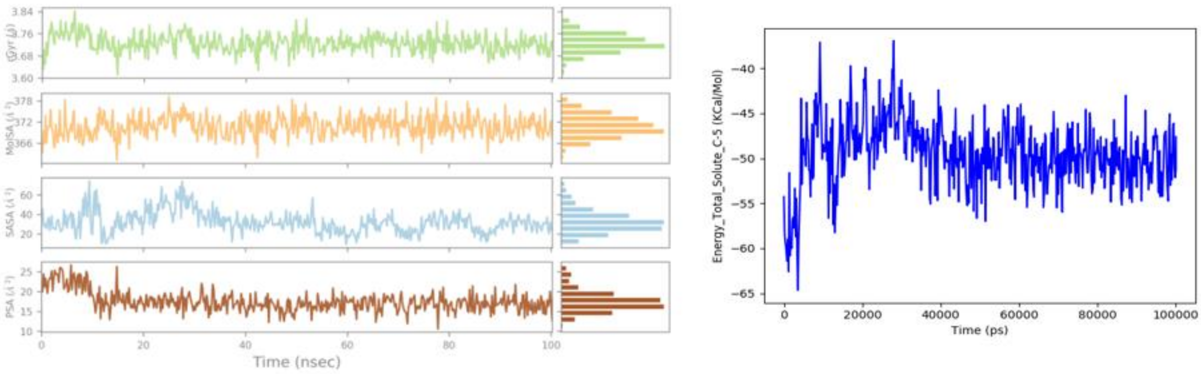

C5

Figure S2: Rg, MolSA, SASA and PSA during 100 ns of MD simulation, and the variation of total free energy for L6, L30, L31 and L37 ligands complexed with DAT protein.
